# Predicting death and lost to follow-up among adults initiating antiretroviral therapy in resource-limited settings: Derivation and external validation of a risk score in Haiti

**DOI:** 10.1371/journal.pone.0201945

**Published:** 2018-08-29

**Authors:** Margaret L. McNairy, Deanna Jannat-Khah, Jean W. Pape, Adias Marcelin, Patrice Joseph, Jean Edward Mathon, Serena Koenig, Martin Wells, Daniel W. Fitzgerald, Arthur Evans

**Affiliations:** 1 Division of General Internal Medicine, Weill Cornell Medicine, New York, United States of America; 2 Center for Global Health, Weill Cornell Medicine, New York, United States of America; 3 Haitian Group for the Study of Kaposi’s Sarcoma and Opportunistic Infections (GHESKIO), Port au Prince Haiti; 4 Division of Global Health Equity, Brigham and Women’s Hospital, Harvard Medical School, Boston MA, United States of America; 5 Department of Statistical Science, Cornell University, Ithaca, United States of America; International Training and Education Center for Health (I-TECH), UNITED STATES

## Abstract

**Background:**

Over 18 million adults have initiated life-saving antiretroviral therapy (ART) in resource-poor settings; however, mortality and lost-to-follow-up rates continue to be high among patients in their first year after treatment start. Clinical decision tools are needed to identify patients at high risk for poor outcomes in order to provide individualized risk assessment and intervention. This study aimed to develop and externally validate risk prediction tools that estimate the probability of dying or of being lost to follow-up (LTF) during the year after starting ART.

**Methods:**

We used a derivation cohort of 7,031 adults age 15–70 years initiating ART from 2007 to 2013 at 6 clinics in Haiti; 242 (3.5%) had documented death and 1,521 (21.6%) were LTF at 1 year after starting ART. The following routinely collected data were used as predictors in two logistic regression models (one to predict death and another to predict LTF): age, gender, weight, CD4 count, WHO Stage, and diagnosis of tuberculosis (TB). The validation cohort consisted of 1,835 adults initiating ART at a different HIV clinic in Haiti during 2012. We assessed model discrimination by measuring the C-statistic, and measured model calibration by how closely the predicted probabilities approximated actual probabilities of the two outcomes. We derived a nomogram and a point-based risk score from the predictive models.

**Findings:**

The model predicting death within the year after starting ART had a C-statistic of 0.75 (95% CI 0.74 to 0.81). There was no evidence for significant overfitting and the predictions were well calibrated. The strongest predictors of 1-year mortality were male gender, low weight, low CD4 count, advanced WHO stage, and the absence of TB. In the validation cohort, the C-statistic was 0.69 (95% CI 0.59 to 0.77). A point-based risk score for death had a C-statistic 0.73 (95% CI 0.69 to 0.76) and categorizes patients as low risk (<2% risk of death), average risk (3–4%), and high-risk (8–10%) and very high-risk (14–19%) with likelihood ratios to be used in settings where the baseline risk is different from our study population. The model predicting LTF did not discriminate well (C-statistic 0.59).

**Conclusions:**

A simple risk-score using routinely collected data can predict 1-year mortality after ART initiation for HIV-positive adults in Haiti. However, predicting lost to follow-up using routinely collected data was not as successful. The next step is to assess whether use of this risk score can identify patients who need tailored services to reduce mortality in resource-poor settings such as Haiti.

## Introduction

During the past decade, over 18 million adults have initiated life-saving antiretroviral therapy (ART) in resource-poor settings [1]. The full benefits of these therapies—improved health for persons living with HIV as well as reduced transmission of HIV to others—will only be realized if patients remain in care and adhere to their medications [2]. The short-term goals during the first year of treatment, therefore, are to minimize those who die and those who are lost from care. Mortality is highest during the first year of treatment in resource-poor settings, even among more recent cohorts with less advanced disease [3–7]. Lost to follow-up (LTF) is also more common during the first year; among all LTF, one-third of patients who become lost do so within the first six months of entering care [7–10].

The WHO 2015 guidelines recommend tailoring care with different care packages of HIV services based on patients’ risk of poor outcomes [11]. For example, patients at highest risk for death may require more intensive or more frequent care as compared to stable patients. Patients who are high-risk for LTF will also need additional interventions aimed at improving retention. Clinically stable patients may be eligible for less intensive community-based care, which requires fewer clinical assessments at health facilities, which in turn relieves overburdened health facilities and increases patient satisfaction [12].

To appropriately and efficiently tailor care for patients at high risk for poor outcomes, there is an urgent need to develop and validate clinical prediction tools that identify patients at highest risk for death and LTF [13]. Ideally, such tools would be applicable when patients first enter care, allowing immediate triage to the services that would most efficiently minimize death and maximize retention in care during the first year. Risk scores have been developed based on data from US and European cohorts to assess short-term disease progression in HIV-positive patients on ART, one-year mortality, and viral suppression, but there are no validated models for predicting the risk of death and LTF for adults newly started on ART in resource-poor settings [14–16].

Our goal was to develop and externally validate practical clinical prediction tools that used routinely collected data from public HIV clinics in Haiti to predict death and LTF over the first year of treatment among adults initiating ART. We believe that if clinics with limited resources could easily use an accurate tool for predicting poor clinical outcomes, then it might help in tailoring services that would optimize the delivery of health care.

## Methods

### Study setting and population

Haiti is the poorest country in the Western Hemisphere and has the highest number of persons living with HIV in the Caribbean, the region most affected by HIV outside of Africa [17]. The prevalence of HIV among adults in Haiti declined from 6.6% in 1993 to 1.8% in 2014 [18]. The Haitian Group for the Study of Kaposi’s Sarcoma and Opportunistic Infections (GHESKIO) is a Haitian non-governmental organization that is the oldest and largest provider of HIV services in the Caribbean, having provided testing since 1985 and treatment since 2003 in the GHESKIO HIV specialty clinic in Port-au-Prince [19]. In 2005, GHESKIO, supported through the President’s Emergency Plan for AIDS Relief, collaborated with the Haitian Ministry of Health to scale-up HIV testing and treatment at 22 clinics across all regions of Haiti. Cumulatively, GHESKIO-supported HIV clinics provide approximately one-third of HIV services for all persons living with HIV in Haiti.

The derivation cohort included HIV-positive adults who received ART in a HIV clinic that had provided ART since 2007, the year clinics began using an electronic medical record (EMR). Inclusion criteria included clinics providing HIV care and ART supported by GHESKIO as an implementing partner and use of an electronic medical record (EMR). We excluded any clinic that had less than 80% accuracy in outcome documentation defined by concordance of vital status documented in two different domains in the EMR where this data was available. The derivation cohort included 6 clinics that met inclusion criteria; all 6 clinics used the Haitian Ministry of Public Health iSanté EMR system [20, 21]. The validation cohort included HIV-positive adults who attended the GHESKIO HIV specialty clinic in Port-au-Prince and received ART from January–December 2012. This clinic is the largest HIV clinic in Haiti and used a separate EMR system [22].

The study subjects in both the derivation and validation cohorts were adults age 15 to 70 years who initiated ART (at least one pharmacy pick-up of triple antiretroviral medications) between 2007 and 2013 in the derivation cohort and during 2012 in the validation cohort. Patients with documented pregnancy at time of ART initiation were excluded. ART initiation was based on WHO guidelines: WHO Stage IV or CD4 count <200 cells/uL (for years 2007–2009), and WHO stage III or IV or CD4 count <350 cells/uL (for years 2009–2013) [23, 24]. From 2007–2009, the first-line ART regimen was zidovudine, lamivudine, and either efavirenz or nevirapine, with single-drug substitutions permitted as outlined by WHO. In 2010, tenofovir-based regimens became first-line treatment.

Clinic visits were routinely scheduled monthly for the first three months and then every three months thereafter. Medications were dispensed monthly. CD4 counts were performed every six months. Viral load was not routinely measured. Retention in care and adherence to therapy were encouraged by peer counseling and social support programs. Field workers attempted to find patients who missed a clinic appointment for up to six months from the date of the missed appointment to ascertain vital status.

### Clinical measurements

Measurements at the time of ART initiation included age, sex, body weight, WHO stage, CD4 count, and diagnosis of tuberculosis (TB). We used the WHO stage and CD4 count that was closest (within 3 months) to the date of initiation of ART. If there was no value within that interval, then we considered the value missing. A diagnosis of TB was defined as documentation of TB in the EMR on the date of ART initiation, or during the prior 6 months. Guidelines for diagnosing TB in Haiti follow the American Thoracic Society and include presence of TB symptoms, chest x-ray suggestive of TB, and either positive microscopy or culture, which is consistent with other studies [5, 25, 26]. Dates of clinic and pharmacy visits were also collected.

Patient information was documented by health care providers on national patient forms and subsequently entered by trained data clerks into the EMR. Data quality assessments were done quarterly comparing paper records to data in the electronic database.

### Outcomes

Individuals were considered dead at 1 year after ART initiation if a date of death within 365 days of ART initiation was recorded in the medical record. Deaths are typically reported to clinic providers by patient families or friends or ascertained by field workers. Haiti does not have a national death registry. We validated this measurement by confirming there were no clinic visits or pharmacy pick-ups after the documented death date. Individuals were considered LTF at 1 year after ART initiation if the individual was not known to be dead and if there was no clinic or pharmacy visit more than 365 days after ART initiation. Individuals with a documented transfer to another clinic were considered alive and not LTF. Patients had a minimum of 2 years of study follow-up from time of the last patient initiation on ART (December 2013) through data of study censor (December 2015).

### Risk score development and validation

In developing and validating a risk score, we followed the recommendations of the 2015 guidelines on the Transparent Reporting of a multivariable prediction model for Individual Prognosis or Diagnosis (TRIPOD) and the modeling strategies of Harrell [27, 28]. For missing data, we applied recommended methods of multiple imputation using bootstrapped samples and chained equations to minimize bias and provide appropriate measures of uncertainty for model estimates [28, 29]. We used 35 bootstrapped imputations based on the rule of thumb of 1 imputation for every percentage point of missing values for the variable with the most missing values [28]. We imputed values for all variables with missing data (including vital status) and, to avoid bias, used the response variable to impute values for missing predictor variables (28, 29). We conducted the imputations with the *aregImpute* algorithm in R, which provides Type 1 predictive mean matching, with weighted selection of donors [30, 31]. Missing vital status was imputed because it was not missing completely at random (26, 46), and therefore dropping those cases from analysis would have biased results. As recommended by von Hippel, we analyzed the data using both the fully imputed dataset and after first imputing all missing values and then deleting those cases with missing outcome data (46). Results from these two approaches are usually the same except for smaller standard errors for the approach of multiple imputation, then deletion (46).

### Sample size

Because the size of the derivation cohort was fixed (n = 7,031) and there were 242 subjects who died in the first year, we limited the number of candidate predictor variables (including nonlinear terms) to 12 (to assure a ratio of deaths to candidate predictors of 20:1 or greater). We excluded interaction terms in this calculation because all interaction terms were evaluated jointly in a single chunk test.

### Model building

We used the logistic regression model to predict death (or lost to follow-up) within 1 year after ART initiation. We avoided automated stepwise variable selection to prevent over-fitting. The three continuous predictor variables—age, weight, and CD4 count—were each represented as restricted cubic splines with 3 knots to account for potentially important nonlinear relationships with the log-odds of the outcome, while limiting expenditure of degrees of freedom by using only 3 knots. We initially entered the three categorical variables—sex, WHO stage, and TB—using their original categories. Because we hypothesized that the effect of weight might be modified by sex and age, we tested the three-way and all two-way interactions among these three variables. No other interactions were tested. Our modeling strategy was to first perform a chunk test of all interaction terms, with the idea of excluding them all if the test was not significant. We next performed a chunk test on all non-linear terms. If this chunk test was significant, then we included non-linear terms for each of the continuous variables. For these chunk tests, we used P<0.2 as a measure of significance [28].

### Internal validation

To measure the magnitude of model over-fitting, we used 200 bootstrap samples to estimate the optimism-corrected indices of model discrimination and calibration [28]. The C-statistic is identical to the area under the ROC curve and measures how well model predictions discriminate between those with and without the outcome of interest. The optimism-corrected C-statistic is always smaller because it estimates how well model predictions will perform if used in a different population (since model predictions are optimized for the derivation cohort).

To visually assess calibration (agreement between predicted and actual probability), we used a loess smoother to plot the actual probability of 1-year mortality as a function of the predicted probability. A curve that maintains a slope of 1 has perfect calibration. When the calibration curve is also corrected for overfitting (bias-corrected) using bootstrapping methods, the slope decreases further, moving the calibration curve further away from the optimal diagonal line, indicating that predictions are less informative when we estimate their performance in other populations [27].

### External validation

For external validation, we tested model performance in a cohort that differed in many respects from the derivation cohort: calendar year of ART initiation, different CD4 count threshold to initiated ART, different clinic site with different providers and staff, different data collection methods, and different electronic health records. These differences allow for a more rigorous test of external validity. We used the C-statistic to compare model performance in the derivation and validation cohorts.

### Nomogram and risk score

The predictive model with its three continuous predictor variables was translated into a nomogram, which is a paper-based tool for easily estimating an individual patient’s risk of dying within the first year. As an alternative tool that is even simpler to use, we created a risk score using the methods of Sullivan and D’Agostino [32]. To produce the risk score, we categorized the 3 continuous risk factors—age, CD4 count, and weight—using cut points determined by visual inspection of their adjusted non-linear relationship to the outcome (S1 Fig). Points for each predictor are summed and a simple table indicates how risk changes for each risk score. We report predicted risks, as well as likelihood ratios, for different point totals. The likelihood ratios allow the risk score to be used even in settings where the baseline risk is different from our study populations; they describe how much each risk score modifies the baseline odds of death.

### Ethics

This analysis was approved by institutional review boards at Weill Cornell Medicine and the Ethics Board at GHESKIO.

## Results

Table 1 reports the characteristics of adults in the derivation (n = 7,031) and validation (n = 1,835) cohorts. In both cohorts, the majority of individuals were female and median age (36–37 years) was similar. In the derivation cohort, 23% of individuals had a missing CD4 count and 35% had missing WHO stage. In contrast, the validation cohort had no missing CD4 count data and only 6% were missing WHO stage. A diagnosis of TB at time of ART initiation was documented for 135 (1.9%) and 228 (12.4%) of adults in the derivation and validation cohorts, respectively.

**Table 1:** Characteristics of HIV-positive adults in the derivation and validation cohorts.

	Derivation Cohort	Validation Cohort
	N = 7031 (6 clinics)	N = 1835 (1 clinic)
**CHARACTERISTIC at time of ART Initiation**		
**Dates of ART initiation**	2007–2013	2012
**Female** n (%)	4439 (63.1%)	1099 (59.9%)
Missing	0 (0)	0 (0)
**Age** Median (IQR) years	37 (30–45)	36 (28–45)
Range	(15–70)	(15–70)
Missing n (%)	8 (0.1%)	0 (0)
**Weight** Median (IQR) kilograms	56 (50–64)	55 (47–62)
Range	(28–90)	(28–90)
Missing n (%)	1044 (14.8%)	114 (6.2%)
**CD4 count** Median (IQR) cells/mm^3^	248 (135–346)	259 (109–356)
Range	(0–2754)	(0–2150)
Missing n (%)	1599 (22.7%)	0 (0)
**WHO Stage** n (%)		
**I/II**	2932 (41.7%)	1430 (77.9%)
III/IV	1639 (23.3%)	291 (15.9%)
Missing	2460 (35.0%)	114 (6.2%)
**Documented Diagnosis TB** n (%)	135 (1.9%)	228 (12.4%)
**ART regimen at initiation**		
AZT/3TC/NVP or EFZ	4871 (69.2%)	220 (12.0%)
TDF/3TC/NVP or EFZ	1460 (20.8%)	1458 (79.5%)
Other	700 (10.0%)	157 (8.5%)
**Vital Status at 1 Year**		
Documented dead n (%)	242 (3.4%)	50 (2.7%)
Alive n (%)	5268 (74.9%)	953 (51.9%)
Missing vital status n (%) (lost to follow-up)	1521 (21.6%)	832 (45.3%)

At 1 year after starting treatment, 242 (3.4%) patients had documented death and 1,521 (21.6%) were lost to follow-up in the derivation cohort. In the validation cohort, 50 (2.7%) patients were dead and 832 (45.3%) were lost to follow-up within 1 year of starting treatment. Mean time to death among the 242 deaths in the derivation cohort was 121 days (standard deviation 6.80, 95% CI 107.45–134.25), and among the 50 deaths in the validation cohort was 103.62 days (standard deviation 22.23, 95% CI 58.95–148.30).

### Prediction model for death

After multiple imputations of missing data, we fit a full logistic model with all three categorical predictors (sex, TB, WHO stage), all three continuous predictors (age, weight, CD4 count), along with 3-knot restricted cubic splines for the continuous variables. The full model’s chi-squared value was 154 (11 df) and therefore the calculated shrinkage factor was 0.93 ([154−11]/154), indicating that the model’s estimated beta coefficients are likely over-optimistic by 7% (low likelihood of model over-fitting).

### Variable selection

A chunk test of the pre-specified 2- and 3-way interactions between age, sex, and weight was not significant (P = 0.68; 12 df) and therefore all interaction terms were dropped. A chunk test of the nonlinear variables was significant (P = 0.0002). The nonlinear components of CD4 count (P = 0.0002) and weight (P = 0.02) appeared to be important aspects of the model. The nonlinear component for age, however, was not significant (P = 0.82), suggesting that the cubic splines associated with age could be dropped from the model.

Although WHO stage was a significant independent predictor (P = 0.007; 3 df), we dichotomized stage into WHO stages I–III vs WHO stage IV, because stages I and III had nearly identical risks of death in the multivariable model, while stage IV was associated with a much higher risk (Table 2).

**Table 2:** Multivariable predictors of 1-year mortality after ART initiation in the derivation cohort (N = 7,031).

Predictor	OR	95% CI	P value
Male sex	2.0	1.5 to 2.7	<0.0001
TB diagnosis	0.3	0.1 to 0.9	0.01
WHO stage†			
I	*reference*		
II	0.6	0.4 to 1.0	0.06
III	1.0	0.6 to 1.5	0.91
IV	2.0	1.0 to 4.1	0.06
WHO stage†			
IV (compared to I-III)	2.2	1.2 to 4.1	0.01
Age (45 vs 30 y)*	1.13	1.0 to 1.4	0.12
Weight (50 vs 64 kg)*	2.3	1.8 to 2.9	<0.0001
CD4 count (135 vs 346 cells/μL)*	1.8	1.5 to 2.3	<0.0001

* OR for interquartile range (75^th^ percentile vs 25^th^ percentile). Because weight and CD4 count have a nonlinear relationship with the outcome, there is no consistent OR for an interval such as 10 kg or 100 cells/μL. The interquartile OR offers a metric for comparing the relative strength of the 3 continuous predictors.

† Because the first three WHO stages had a similar risk of death in the multivariable model, and were not statistically distinct, we collapsed WHO stages I-III.

The final model therefore included all 6 predictors, with CD4 count and weight needing restricted cubic splines to represent their nonlinear relationship with the outcome. The effects of each of the continuous predictors on the predicted probability of dying before 1 year, adjusting for all other variables in the model, are provided in S1 Fig.

Table 2 describes the magnitude of the effect of each variable on the odds of being dead at 1 year after ART initiation. For the continuous variables, we calculated an odds ratio comparing the 25^th^ percentile to the 75^th^ percentile of the predictor variable as a way to compare the relative importance of predictors (26). The four strongest predictors of death in the first year had similar predictive values (each approximately doubling the odds of dying in the first year): male sex, WHO stage IV, low weight, and low CD4 count. For individuals with a diagnosis of tuberculosis, the odds of dying in the first year was reduced by 70 percent.

**Fig 1:**
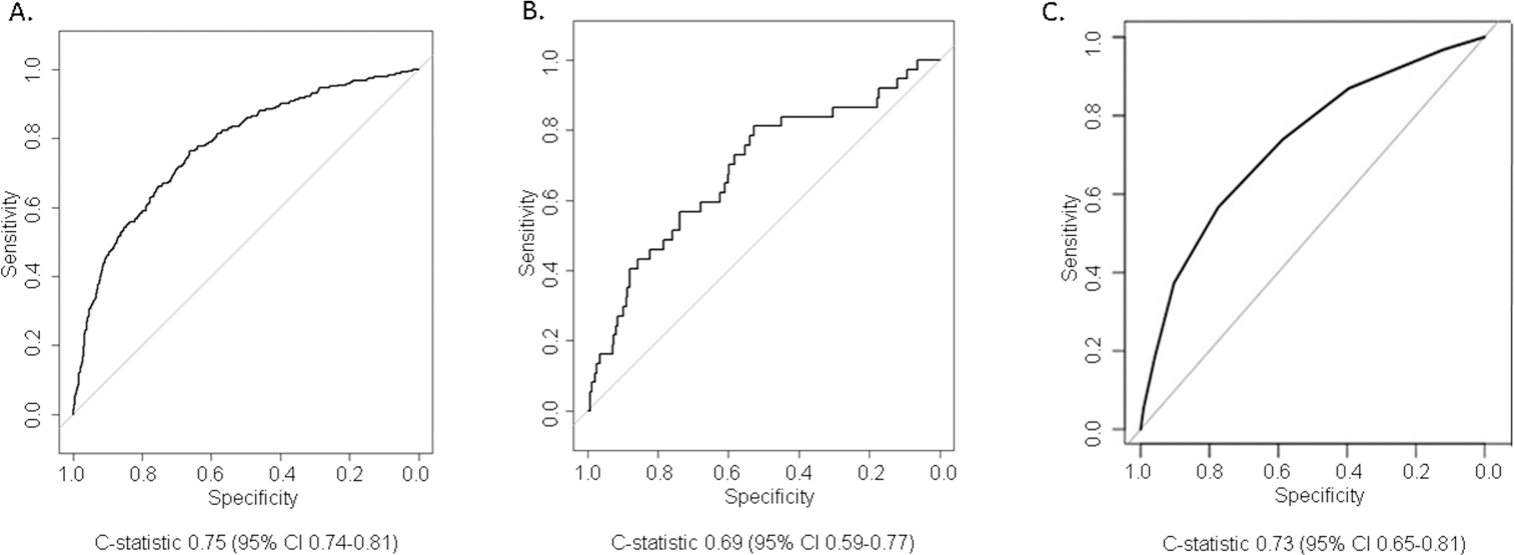
Receiver operator characteristic curves of model predictions of mortality within 1 year of starting ART in the derivation cohort (A), validation cohort (B), and when a simplified risk score is used in the derivation cohort (C).

Fig 1 describes the ROC curves for model predictions in the derivation cohort. The C-statistic was 0.75 (95% CI 0.74–0.81), which means that the model’s risk score is 75% accurate in discriminating between a randomly selected subject who dies in the first year from a randomly selected subject who survives the first year.

Because a diagnosis of TB had an unexpected protective effect on mortality, we explored a model that excluded TB. The C-statistic for that reduced model was nearly identical, 0.749, with an identical Brier score, 0.040, and very similar coefficients for all other variables. This suggests that a risk score without TB would work equally well. However, we only report the model that complies with our pre-specified analytic strategy.

### Internal validation

**Fig 2:**
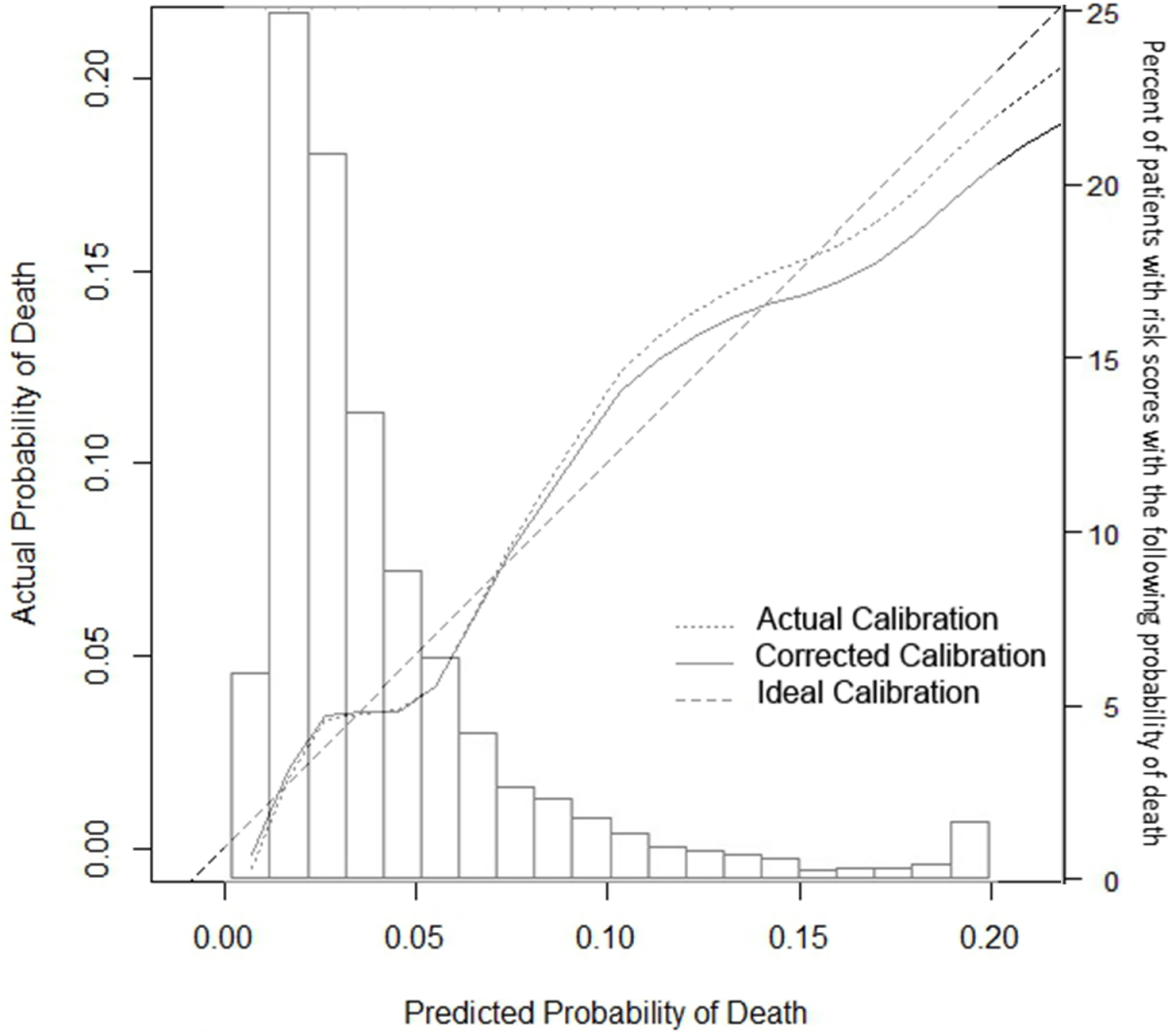
Histogram of the distribution of predicted probability of death in the derivation cohort and superimposed calibration curve describing the relationship between predicted and actual risk of death in the year after starting ART.

Using 200 bootstrap resamples, we estimated model “over-optimism” by measuring the slope of the calibration curve and a bias-corrected area under the ROC curve (26). The calibration curve slope was 0.90, suggesting modest over-fitting that that would require shrinking model coefficients by 10% to improve calibration (Fig 2). After correcting for the over-optimism, the C-statistic decreases from 0.75 to 0.73, meaning that if the risk score is used in future cohorts, there is a 73% probability that a randomly selected individual who dies in the first year after ART initiation will have a higher predicted probability of dying than a randomly selected individual who does not die in the first year.

Inspection of the calibration curve (Fig 2) demonstrates that predictions are less accurate when predicting a 15% or greater chance of death in the first year after starting ART. These predictions are likely to be 2 or 3 percentage points too extreme, because of model over-fitting.

For the calibration curve, the vertical axis is estimated actual probability (using a loess smoother) and the horizontal axis is the model’s predicted probability of 1-year mortality. The ideal calibration curve is represented by the dashed diagonal line with a slope of 1, indicating perfect agreement between predicted and actual probabilities. The model’s actual performance is represented by the dotted line. The solid line is the calibration curve corrected for the bias associated with model overfitting (over-optimism), based on model performance in 200 bootstrapped samples, which approximate the model’s performance in a different population. On average, the model’s predictions differed from actual results by less than 1 percentage point (mean absolute error 0.6 percentage points) and 90% of the differences were less than or equal to 1.2 percentage points. The actual and corrected calibration curves indicate that predicted probabilities greater than15% mortality were overestimated, but predictions less than that were accurate. The histogram and corresponding right-side axis report the percent of subjects with risk scores with the corresponding probability of death within the first year after ART initiation.

### External validation

The model’s performance in the validation cohort yielded a C-statistic of 0.69 (95% CI 0.59–0.77) (Fig 1C), which was not statistically different from the C-statistic in the derivation cohort.

### Sensitivity analysis

After following von Hippel’s recommendation to first multiply impute missing data for all variables (predictors and outcome) and then delete all observations that originally had missing outcome values, we built a model on the reduced derivation sample (5510 subjects with known vital status, including 242 deaths) [33]. The C-statistic for the model was comparable, 0.755, and none of the coefficients, or their confidence intervals (data not shown), were appreciably different from the model results on the full derivation sample.

### Model risk prediction

Table 3 describes the point-based risk score that predicts an individual’s risk of death in the first year of treatment. The simplified scoring rule is somewhat less discriminating, with a C-statistic in the derivation cohort of 0.73 (95% CI 0.69 to 0.76) that is smaller than for the full model (0.75), which did not categorize the 3 continuous variables. For example, a 55 year old man, who weighs 58 kg, has a CD4 count of 50 cells/mm3, has not been diagnosed with TB, and is WHO stage IV has a risk score of 9, which corresponds to a risk of death of 14–19%, approximately 4 times higher than the baseline odds of death.

**Table 3:** Simplified risk score for death within the first year after ART initiation (A) and corresponding risk associated with each point total (B).

**A.**				
**Variable**	**Category**	**Points**		
**Sex**	Male	1		
	Female	0		
**Weight** *(kg)*	<50	3		
	50–60	1		
	>60	0		
**Diagnosis of Tb**	No	2		
	Yes	0		
**WHO stage**	4	2		
	1–3	0		
**CD4 count** *(cells/*μ*L)*	≤50	3		
	51–100	2		
	101–250	1		
	>250	0		
B.				
Point Total	Risk	Likelihood Ratio	**# deaths/# persons with score** Derivation Cohort	**# deaths /# persons with score** Validation Cohort
0–3	< 2%	0.3	43/2674	11/695
4–5	3–4%	0.5	93/2706	27/628
6	8–10%	1.6	61/930	23/264
7 and higher	14–19%	4	100/721	47/248
Total			297/7031 (5%)	108/1835 (6%)
C-statistic (95%CI)		0.73 (0.69, 0.76)	0.70 (0.62, 0.78)	

Note: For all patients with missing data, including data on vital status at 1 year, values were imputed with chained equations in 35 bootstrapped samples. The data in the last two columns represent the values of one of the 35 imputed datasets which had a mortality closest to the mean mortality over al imputed datasets. No subjects had scores of 10 or 11. Likelihood ratios are the ratios of the probability of a point total among those who died over the probability of the same point total among those who lived. Likelihood ratios represent how much the overall baseline odds of dying changes with a particular point total. For example, a point of 7 means that the odds of dying are 3 times higher than the baseline (total population) odds of dying.

Alternatively, a nomogram for predicting risk of death within the first year after starting ART is available in S2 Fig. Values for each of the six predictors can be assigned points by extending a vertical line from the value of the predictor to the horizontal bar at the top of the nomogram. After summing points for all predictors, the risk of death is estimated by extending a vertical line from the total points bar to the risk bar at the bottom of the nomogram.

### Prediction model for lost to follow-up after ART initiation

The multivariable logistic model predicting lost to follow-up had a C-statistic of 0.59 (95% CI 0.58 to 0.61) (P<0.001), indicating weak ability to discriminate between those who will remain in care from those who will be lost. The significant independent predictors were young age and high WHO stage. Gender, CD4 count, and TB diagnosis were not independent predictors. For example, a 20 year-old subject was twice as likely to be lost to follow-up compared to a 45 year-old; and someone with WHO stage IV was twice as likely to be lost compared to someone with stage I or II (keeping all other variables constant).

Although statistically significant, we felt that the model’s ability to predict lost to follow-up was not sufficiently robust to be clinically useful. Therefore, we did not perform further model validation.

## Discussion

We derived and validated a practical clinical prediction model to estimate the risk of death in the first year after starting ART, a time of high mortality among persons living with HIV in resource-poor settings like Haiti. The risk score performed well in two distinct cohorts and over two time periods, using routinely collected patient data. Risk of death can be estimated with simple addition, which allows the tool to be used across diverse HIV programs in resource-limited settings. By easily risk-stratifying patients on initial presentation, healthcare resources can be better allocated to improve outcomes.

These results provide a novel addition to other HIV-specific risk scores designed to improve the efficiency and cost-effectiveness of HIV prevention and treatment programs. To date, the majority of scores for use in resource-limited settings have focused on predicting the risk of HIV acquisition among women or among HIV-serodiscordant couples [34–36]. One score has been developed to predict risk of attrition among post-partum HIV-positive women in Zambia, but has not been validated [37]. While efforts to predict ART treatment failure have been done for ART patients in Haiti, this is one of the first proposed clinical decision tools designed to identify high-risk patients at risk of death at time of ART initiation [21].

Our score performs similarly to a risk score for mortality among HIV-positive adults in North America—the Veterans Aging Cohort Study (VACS) Index—which has been advocated for use in clinical programs and research [14, 38, 39]. The VACS Index uses the variables of age, CD4 count, HIV-1 RNA, hemoglobin, tests of liver and renal function, and presence of hepatitis C co-infection to predict death up to six years after ART initiation. The C-statistic of the VACS Index was 0.78 in the derivation cohort, comparable to our model’s C-statistic of 0.75.

Five of the six predictors in our model—male gender, advanced age, low weight, low CD4 count, and advanced WHO stage—have been previously associated with HIV-related mortality [4, 8, 40]. Interestingly, a diagnosis of tuberculosis, the sixth predictor, was associated with lower mortality in the first year after treatment in our model. This finding has several possible explanations. First, a diagnosis of TB at time of initiation of ART might be a marker of early diagnosis of TB, as opposed to delayed or missed diagnosis. This is consistent with studies from Haiti reporting lower mortality among those on TB treatment when ART was initiated compared to those diagnosed and treated for TB after starting ART, particularly in the first three months after treatment start [19, 41]. A study from South Africa provided intensive TB screening for HIV-positive patients starting ART, regardless of symptoms, with AFB smear, tuberculosis polymerase chain reaction, and mycobacterial culture, and found that 19% (148/825) had active infection [42]. In our derivation cohort, only 1.9% had documented TB at time of ART start which suggests that even in a setting with a different TB incidence, likely many patients had a missed or delayed TB diagnosis. A second possible explanation is that a TB diagnosis is a proxy for better engagement in care. TB-infected patients must be retained in care long enough to receive a diagnosis, which requires multiple visits for sample collection and processing. A third explanation is that those with a TB diagnosis include individuals who sought care from the GHESKIO clinics, or were referred by their physicians, because of its outstanding national reputation for treating TB–HIV co-infection. Thus, the clinic attracts the most informed and motivated co-infected patients. However, in contrast to our finding that a TB diagnosis is a strong predictor of survival, other studies have reported that a diagnosis of TB is associated with increased mortality [43, 44].

Since TB diagnosis is likely confounded by favorable unmeasured factors such as engagement in care and better health-seeking behaviors, the risk score needs to be further validated across geographically diverse settings and populations.

Ideally, an HIV program could use this risk score to identify patients at highest risk of early death after starting treatment and provide such patients with differentiated models of HIV care and interventions to reduce or prevent death. For example, a third of deaths in the derivation cohort had a simple score of ≥ 7 total points (Table 3), conferring an odds of death several times higher than baseline, equivalent to an absolute risk of 12% or higher in the first year. Differentiated models of HIV care include altering the types of services offered (e.g., more intensive case management, food support, or prophylactic medications), the location of services (e.g., brief hospitalization), and or the frequency of services (e.g., more frequent visits that are clinic and/or home-based) [45]. Patients with a score of ≥7 points could be offered one or more of these differentiated models of care, and if effective, the overall mortality of the population might be reduced by a third.

Our score is innovative in that is uses routinely collected patient data available in many HIV programs in resource-limited settings, which allows for the score to be used across diverse settings. The score is pragmatic in that it is a simple point-based score that can be calculated by various health care cadres ranging from providers to peer educators, and can be easily adapted to mobile-phone technology, included as an automated tool within an existing EMR, or used on paper.

A limitation of this study is that the risk score is based on data from only one country, Haiti, which differs from other resource-limited settings in health infrastructure, human resources, and HIV prevalence. Additionally, the score was constructed using HIV program data that had a large proportion of missing data, including missing vital status at 1-year follow-up. Developing a risk score in a cohort with complete mortality data, although ideal, is an unrealistic expectation for many public HIV programs in developing countries. Another limitation of our study is that we were not able to identify a robust model that predicts lost to follow-up in the first year of treatment. There are several possible reasons why we could not meet this goal. First, we recognize that the category of lost to follow-up is a heterogeneous group of outcomes: undocumented deaths, undocumented or silent transfers, and individuals who simply disengage from care [46]. Second, a robust prediction of LTF likely depends less on biologic variables, such as age, sex, weight, CD4 count, and TB infection, and more on psychosocial, cultural, and economic variables, which are currently not routinely collected in many public HIV programs in resource-limited settings [7, 25]. Third, accurate prediction of retention in care is probably best done by measuring intermediate outcomes—keeping clinic appointments and taking prescribed medications—rather than variables measured at baseline, when ART is started [47]. Finally, in the era of universal treatment, some HIV programs no longer perform CD4 counts as treatment is available to all patients regardless of their immune status. However, other HIV programs continue to use CD4 counts to prioritize ART among those who are most vulnerable. The proposed risk score uses CD4 counts at time of ART and thus, in the current form is only applicable for HIV programs who have available resources for CD4 testing. Further research can differentiate if a risk score without CD4 counts has similar predictive ability.

An important next step is to evaluate the risk score’s impact on health outcomes. A beneficial impact requires both that the risk score be used and that its use changes behavior [48]. For example, patients identified as high-risk might receive more intensive services to enhance treatment adherence, which has been associated with reduced early mortality [49, 50].

## Conclusion

Over the past decade, the scale-up of ART across resource-limited settings has saved millions of lives. However, too many persons living with HIV still die. Clinical decision tools, such as the validated risk score we describe, are needed to help tailor services to efficiently improve retention and reduce mortality.

## Supporting information

S1 Dataset De-identified dataset. (DTA)

S1 Fig Probability of death during the first year after ART initiation by age, weight and CD4 count. (DOCX)

S2 Fig Nomogram for predicting risk of dying within 12 months after starting ART using values of 6 predictor variables. (DOCX)
