# Supplementary material for: Predicting death and lost to follow-up among adults initiating antiretroviral therapy in resource-limited settings: Derivation and external validation of a risk score in Haiti
Source: PLoS One. 2018 Aug 29;13(8):e0201945. doi: 10.1371/journal.pone.0201945 (PMC6114504; doi:10.1371/journal.pone.0201945)
Supplement: S2 Fig — (DOCX) [file pone.0201945.s003.docx]

Supplement 2.



Nomogram for predicting risk of dying within 12 months after starting ART using values of 6 predictor variables.

*Instructions for using nomogram: 1) Make a mark on the horizontal axis for each patient characteristics. 2) Draw a vertical line up from the mark made at each characteristic to the top line labelled “Points” to calculate the number of points assigned to each patient characteristic. 3) Add the number of points for all characteristics on the line labeled “Total Points.” 4) Draw a line from the mark of total points to the bottom line illustrating the probability of death. Of note, if the total points are < 60, then the patient has a < 1% probability of death; if total points are > 150, then the patient has a predicted probability of death of > 20%.*


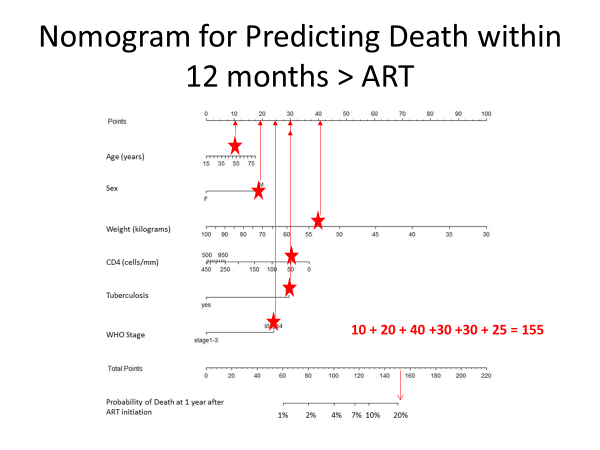
Nomogram with example patient: A 55 year-old male, weighing 53 kilograms, with CD4 count of 50 cells/mm^3^, no diagnosis of TB at time of ART initiation, and WHO Stage 4. The patient received 10 points for age, 20 points for sex, 40 points for weight, approximately 30 points for CD4 count, 30 points for absence of TB, and 25 points for WHO Stage. His point total is 155 which corresponds to a probability of death of approximately 20% after one year of ART initiation.
