# Supplementary material for: Predicting death and lost to follow-up among adults initiating antiretroviral therapy in resource-limited settings: Derivation and external validation of a risk score in Haiti
Source: PLoS One. 2018 Aug 29;13(8):e0201945. doi: 10.1371/journal.pone.0201945 (PMC6114504; doi:10.1371/journal.pone.0201945)
Supplement: Supplementary file 2 [file pone.0201945.s002.docx]

Supplement 1: Probability of death during the first year after ART initiation by age, weight, and CD4 count, adjusting for all variables in model (age, sex, weight, diagnosis of TB, and WHO Stage).

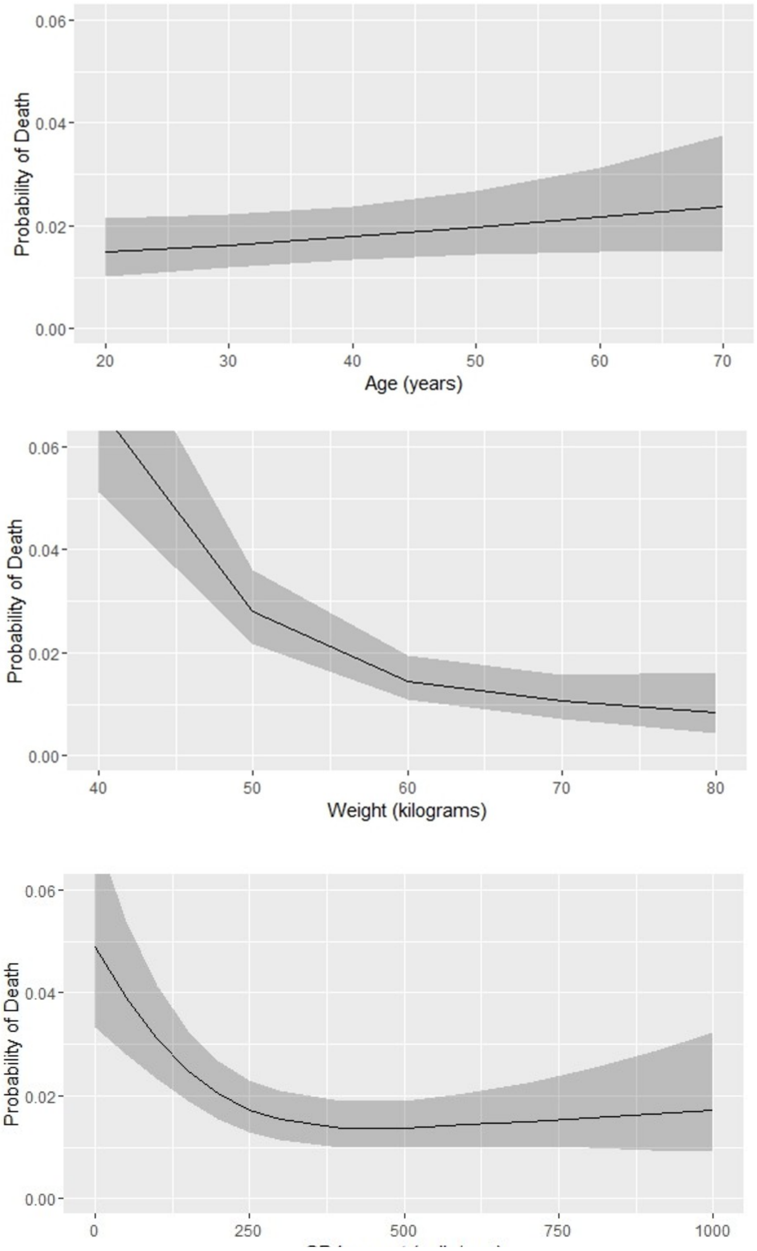

CD4 count (cells/uL)
